# Diurnal Variation in Medical Emergency Team Calls at a Tertiary Care Children’s Hospital

**DOI:** 10.1097/pq9.0000000000000341

**Published:** 2020-09-07

**Authors:** Susan R. Conway, Ken Tegtmeyer, Derek S. Wheeler, Allison Loechtenfeldt, Erika L. Stalets, Patrick W. Brady

**Affiliations:** From the * Division of Critical Care Medicine, Children’s National Hospital, Washington, D.C.; † Department of Pediatrics, George Washington University School of Medicine, Washington, D.C.; ‡ Division of Critical Care Medicine, Cincinnati Children’s Hospital Medical Center, Cincinnati, Ohio; § Department of Pediatrics, University of Cincinnati College of Medicine, Cincinnati, Ohio; ¶ Division of Critical Care Medicine, Lurie Children's Hospital of Chicago, Chicago, Ill.; ‖ Department of Pediatrics, Northwestern University Feinberg School of Medicine, Chicago, Ill.; ** Division of Hospital Medicine, Cincinnati Children’s Hospital Medical Center, Cincinnati, Ohio

## Abstract

Medical emergency teams (METs) bring critical care expertise to the bedsides of hospital ward patients who may be deteriorating. Diurnal variation in MET activation rates may identify inconsistencies in the detection of patients needing intervention. We aimed to determine whether such variation exists at our tertiary care children’s hospital.

**Methods:**

In this retrospective cohort study, we collected data including date and time of MET and disposition following MET for all inpatients at Cincinnati Children’s Hospital Medical Center with a MET call between January 2008 and May 2014. The analysis compared the MET rate between days and nights, weekdays and weekends, and before and after nursing shift change.

**Results:**

The number of METs per hour varied throughout the day. More METs were called during the day than at night (0.7 calls/shift ± 0.95 vs 0.6 ± 0.9, *P* < 0.001). There were also more METs per day on weekdays than weekends (1.4 ± 1 calls/d vs 1.2 ± 1, *P* < 0.001). Daytime METs were more likely to lead to transfer to the intensive care unit or operating room than those called at night (61.9% vs. 52.9%, *P < 0*.001). MET activation rates did not differ significantly in the 2 hours before nursing shift change compared to the 2 hours after.

**Conclusions:**

At our large tertiary care children’s hospital, there are both diurnal variations and variations across weekdays versus weekends in the MET activation rate. This difference may indicate variations in our ability to detect deteriorating patients on the wards and be further studied.

## INTRODUCTION

Medical emergency teams (METs) bring critical care expertise to the bedside of deteriorating patients on hospital wards. Over the past 15 years, METs have been instituted in hospitals nationwide and have decreased mortality and cardiopulmonary arrest rates outside of the intensive care unit (ICU).^1–6^ Hospitals that have established a MET as part of a rapid response system often adopt a set of clinical warning signs to help identify deteriorating patients who might benefit from a MET.^7–9^ Nurse or physician concern, and often parental or family concern, may also be a cause for MET calls.^10^ The detection of patients who might be deteriorating is an essential component of a rapid response system.^11^

Several studies in hospitals that care primarily for adults have identified variation in the rate of MET calls across days versus nights and weekdays versus weekends.^12–14^ Although the reason for the difference in calls is not well understood, several investigators have suggested that it might correlate with increased monitoring. Perhaps, deteriorating patients are identified more often during the day and on weekdays, when staffing is better, and monitoring may be more robust.^12,13^ More limited research conducted in children’s hospitals suggests a predominance of MET calls during the day.^15–17^ These studies were complicated by the inclusion of codes and other emergent events in the analysis^15^ and the relative infrequency of MET calls (25% of ICU transfers).^16^ No study has been designed and powered to examine diurnal variation in MET calls at a children’s hospital with a well-established MET system.

At our tertiary care children’s hospital, we have had a MET system in operation since 2005; it has been associated with a decrease in codes outside of the ICU.^1^ We identify deteriorating patients on the wards using a Pediatric Early Warning Score (PEWS), along with nurse, physician, or family concerns.^7^ In this report, our objective was to compare the rate of MET calls across days and nights and weekdays and weekends to identify potential lapses in the detection limb of our rapid response system. We hypothesized that we would find an increased rate of MET calls during the day and on weekdays, corresponding to the increased staff availability.

## MATERIALS AND METHODS

### Study Context

We conducted the study at Cincinnati Children’s Hospital Medical Center (CCHMC). This tertiary care children’s hospital serves as a regional referral center for the Midwestern United States and a community hospital for the greater Cincinnati area. At the time of the study, the hospital had 482 inpatient beds, including a 35-bed pediatric ICU (PICU), a 25-bed cardiac ICU (CICU), and a 59-bed neonatal ICU (NICU). Patient care teams include a bedside nurse, the floor charge nurse, and a physician team that is generally comprised 3–4 interns, 1–2 senior residents, a fellow, and an attending physician. The patient-to-nurse ratios increase by approximately 50% on nights and weekends. On nights, residents often function without support from an in-house fellow or attending, who commonly take night-call from home. The number of residents in the hospital generally also decreases by 50%–75% on nights and weekends. Nurses on the floor work 8- or 12-hour shifts, and potential shift changes occur at 7:00 am, 3:00 pm, 7:00 pm, and 11:00 pm.

The MET includes the floor team caring for the patient, the hospital nurse manager, and the responding PICU-based team—fellow, resource nurse, and respiratory therapist. When a MET is called, the PICU team has a maximum of 15 minutes to arrive at the patient’s bedside, although internal audits show an average response time of approximately 7 minutes. The group then collaborates to determine the best management plan for the patient. If the team determines that the patient would benefit from the escalation in care, they transfer the patient, and the ICU team assumes primary responsibility for the patient. Historically, 50%–60% of MET calls result in transfer to the ICU at our institution. Our emergency response system includes the Code Blue, activation of which brings the hospital-wide code team to the patient’s bedside as quickly as possible. Transfer from the hospital floor to the ICU at CCHMC requires emergency response activation, either Code Blue or MET. Criteria for MET calls include nurse, physician, patient, or family concerns. The PEWS is designed to support clinical decision-making regarding the need for escalation in care but is not a trigger for a MET call. When a patient has a total PEWS of 7 or above, clinical decision support in the electronic health record alerts the nurse that a MET should be called. In our experience, however, nurses commonly choose to alert the team and ask for a bedside assessment but do not ask for a MET to be called solely based on the PEWS. If a MET call does not result in a transfer, hospital culture encourages members of the MET to regroup at a prespecified time—often 1–2 hours following the initial call—to reassess and determine whether the patient’s disposition remains appropriate. Our quality improvement work aims to empower all bedside team members to call a MET if they have concerns about a patient and express those concerns when the MET meets.

### Data Collection

We collected data retrospectively from a secure database of all METs called between January 2008 and May 2014. All patients requiring a MET during this period were included in the study, with no additional inclusion or exclusion criteria. Data obtained included patient age and gender, unit location, date and time of MET call, and disposition following the call. Daytime was defined as the period from 7:00 am to 6:59 pm; nighttime was defined as the period from 7:00 pm to 6:59 am. Disposition following the MET call was a dichotomous variable defined as either continuation of care on the floor or escalation of care—transfer to the PICU, NICU, CICU, or operating room (OR). If the transfer occurred within 2 hours of MET call, disposition was considered an escalation of care. If the transfer occurred more than 2 hours after MET call, even later on the same day, then disposition was considered a continuation of care on the floor.

### Data Analysis

To determine the difference in MET call rates during the day and night, we first divided the 24-hour day into 1-hour intervals. We graphically displayed the absolute number of MET calls that occurred over each 1-hour block for the entire 6-year study interval, dividing each total number by the number of months in the study period to give METs called per hour per month. We then calculated an average and SD for the number of MET calls during the 12-hour day and night. We then compared these averages using Student’s t-test. Similarly, to determine the difference in the rate of MET calls on weekdays versus weekends, we calculated the average and SD for the rate of MET calls on weekdays and weekends. We compared them using Student’s t-test.

To look for a possible effect of nursing shift changes on the rate of MET calls, we examined the average rate of MET calls before and after a shift change. Specifically, we compared the average rate of MET calls in the 2 hours before a potential nursing shift change with the rate of METs called in the 2 hours following a potential nursing shift change.

To determine the difference in disposition following a MET activation, we measured the total number of MET calls resulting in escalation versus continuation of care on weekdays and weekends, and days and nights, and calculated the percentage of MET calls at these times resulting in an escalation of care. We compared disposition on weekdays versus weekends and days versus nights using the chi-square test.

## Results

**Fig. 1.**
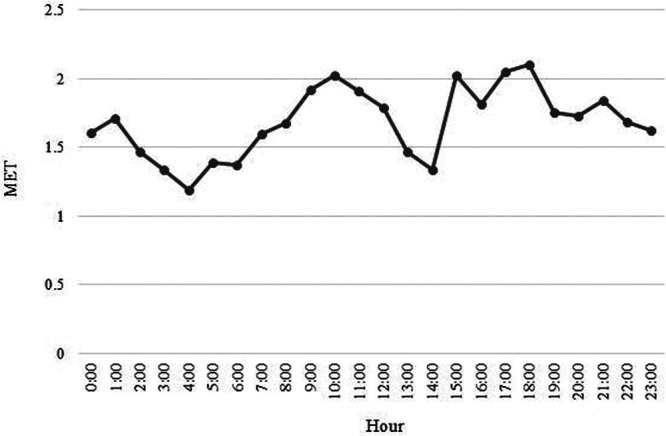
Number of METs called per hour per month over the 24-hour day throughout the 6-year study period. Each hour plotted represents the one-hour block starting at that hour. For example, 1 am represents all METs called per hour per month from 1 am to 1:59 am.

Over our 2,313-day study period, there were 1,652 weekdays and 661 weekend days. During this period, there were a total of 3,115 MET calls. Of these, 1,442 were at night, and 1,673 were during the day. There were 2,337 METs on weekdays and 778 on weekends. There was no significant difference in age or gender of patients who had METs called during the day versus night or on weekends versus weekdays (Table 1). The number of MET calls per hour varied throughout the day (Fig. 1). There was a statistically significant increase in the rate of MET calls during the day versus night, with the average rate of MET calls during the day being 0.7 [SD 0.95, 95% confidence interval (CI) 0.68–0.77] per 12-hour day, whereas the average rate of MET calls at night was 0.6 (SD 0.9, 95% CI 0.58–0.67) per 12-hour night (*P* < 0.001). There was also a higher rate of MET calls on weekdays than weekends, with the average number of MET calls per 24 hours on weekdays 1.4 (SD 1.4, 95% CI 1.36–1.47) and on weekends 1.2 (SD 1.3, 95% CI 1.08–1.27) (*P < 0*.001).

**Table 1.** Gender and Age of Patients with MET Calls, Stratified by Time of Day (Day vs Night) and Day of the Week (Weekday vs Weekend)

	Day	Night	Weekday	Weekend
Gender				
Male	714 (44%)	629 (44%)	1294 (56%)	434 (57%)
Female	926 (56%)	802 (56%)	1009 (44%)	334 (43%)
Age				
<2 y	485 (30%)	431 (30%)	676 (29%)	240 (31%)
2–12 y	718 (44%)	644 (45%)	1031 (45%)	331 (43%)
13–18 y	261 (16%)	199 (14%)	333 (15%)	127 (17%)
>18 y	171 (10%)	147 (10%)	252 (11%)	66 (9%)

Those METs called during the day were significantly more likely to lead to an escalation of care than those called at night (1037 or 61.9% escalation during the day versus 763 or 52.9% escalation at night, *P < 0*.001). On weekends, 60.3% of MET activations (468 events) resulted in escalation to a higher level of care versus 56.9% on weekdays (1328 events); this difference did not reach statistical significance (*P* = 0.10).

To examine the potential effect of nursing shift changes on the MET call rate, we compared the average rates of MET calls before and after nursing shift change. The proportion of all MET calls in the 2 hours following nursing shift change (34.3%) and the proportion in the 2 hours before nursing shift change (32.8%) were similar (*P = 0*.4).

## DISCUSSION

We found a modest but significant difference in the rate of MET activation between days and nights and between weekdays and weekends. Specifically, we show that at our tertiary care children’s hospital, METs are activated more frequently during the day than at night and more frequently on weekdays than on weekends. Over a month, the average rates would be ~22 MET calls during the day and ~19 calls at night. MET activation pattern suggests changes in our detection of deteriorating patients from day to night and weekdays to weekends. The fact that staffing is lower overnight and on weekends, when MET activation is decreased, supports this idea.

Perhaps, the most obvious factor contributing to our ability to detect and intervene upon deteriorating patients on the wards is patient monitoring. This monitoring includes regular nursing assessments and PEWS and parental observation and physician team assessments. Others have shown that parental concern and a “gut feeling” by a clinician often correlate with the risk of deterioration.^6–9^ Both would likely be decreased at night when parents often sleep, and individual physicians are responsible for many patients. If staffing and monitoring decrease patient interaction at night and contributes to decreased detection of deterioration at night, further studies should address how to maintain consistency in the MET system’s detection limb.

On the other hand, it is not necessarily true that a decreased MET activation rate represents a failure to detect patient deterioration, as has previously been noted.^13^ An important possibility is that patients are detected but not intervened upon (ie, no MET is called for some other reason). At our hospital, we have worked to improve communication between bedside nursing staff and physician teams using inpatient huddles, unit huddles, and “robust plans” for high-risk patients.^9^ Still, it is possible that the environment overnight, in which physicians and nurses may feel more overwhelmed or less supported, causes decreased situation awareness and communication between members of the care team. This possibility could make it less likely that a MET would be called, even in the setting of concern on the part of one or more members of the care team. There is also potential that teams recognizing lower staffing at night pre-emptively activate METs during the day shift, rather than letting lingering concerns have further opportunity to deteriorate. Last, it is possible but perhaps less likely that the patient census during the day has higher acuity or complexity than the patient population remaining in the hospital overnight.

Previous studies have shown a gradual increase in the MET call rate in the morning, beginning at the time of morning handover and physician rounds.^12,13^ Scheduled nursing handovers have also been peak times for MET calls, as compared with the average MET call rate the rest of the day.^12^ We saw a gradual increase in MET call rate starting from approximately 6 am, when resident teams begin their morning handover, through 10 am, generally midway through morning rounds (Fig. 1). Nursing shift changes at our hospital are a time (day or night) when nurses review their patients and go to the bedside for routine handover. We hypothesized that these might be times when deteriorating patients who were thus far undetected would be noticed and might cause a spike in the MET call rate. However, we did not see a significant effect of nursing shift changes on the MET call rate.

In addition to diurnal variation in the rate of MET calls, our data show a corresponding variation in disposition following a MET call. A higher proportion of MET activations during the day results in an escalation of care. We might hypothesize that some of this difference is due to the spike in MET calls that we see in the early morning through morning rounds. These METs may be called on patients who went unrecognized throughout the night. An increased proportion of them may require an escalation in care. Alternatively, it may be that faculty physicians’ greater presence during the day results in more successful advocacy to transfer intermediately an ill patient to the ICU. However, our hospital default is that unless there is team consensus on staying, the patient will transfer to the ICU.

Further work will need to be done to understand this diurnal variation in MET disposition. If a higher proportion of patients require transfer to an intensive care setting, we may be close to missing our window of intervention, thus increasing the likelihood of preventable code events or delayed transfer to the ICU. To test this hypothesis in our future work, we will need to correlate the transfer time to the ICU with the severity of illness scores and the need for invasive ventilation or pressor support upon transfer.

This study has several limitations. We cannot conclude the causes of diurnal variation in MET rates based on this retrospective observational study. Second, we used data collected over 6 years to identify patterns in MET activation rates. There were changes over this time in characteristics of our MET system, along with hospital and ICU bed capacity, and the typical acuity of patients on the wards that may have led to changes in the MET activation patterns over time, potentially confounding our data. One notable change in the fall of 2009 was our elimination of the “curbside” PICU consult and the development of a system of proactive identification of watcher patients. These changes led to a special cause change in the rate of our MET calls from a median of 8–40 calls per month.^9^ We also attempted to examine the effect of nursing shift changes using hospital-wide nursing shift change times. However, we did not have patient-specific data on when nurses caring for particular patients were changing shifts. This deficiency may have limited our ability to detect an effect of nursing shift change on MET activation rates.

Similarly, residents often begin morning hand-off before 7 am, so that in some cases the daytime residents may have assumed care of a patient before our defined day-night cutoff was reached. Additionally, although we did not appreciate differential bed availability between days and nights, we did not have access to bed availability to control for this variable. Last, data from our tertiary care children’s hospital may not generalize to other hospitals nationwide with different patient profiles and staffing capacities or MET criteria.

Our hospital instituted a “watcher” system in 2009 to create situation awareness among providers caring for patients felt to be at increased risk for deterioration. Plans are made for these patients that include specific criteria for which a MET will be called. We have more recently implemented “sepsis huddles” that aim to establish a mental model and expedite treatment for septic patients. We might expect such interventions to improve the detection of sicker ward patients regardless of staffing changes and decrease diurnal variation in the MET rate. As quality improvement work continues, it may be useful to examine its effect on the baseline diurnal variation that we have identified here. Also, it may be useful to examine seasonal changes in MET call rates and outcomes, as patient acuity, hospital census, and trainee experience vary significantly throughout the year. Last, broadening these analyses to include multiple children’s hospitals may aid our ability as a community to develop robust systems for identifying and expediently treating the sickest patients on our hospital wards.

## CONCLUSIONS

We have shown diurnal variability in the rate of MET activation at our tertiary care children’s hospital. This variation indicates a possible decrease in the detection of deteriorating patients in our system overnight and on weekends, perhaps correlated with decreased staffing. It will be essential to analyze this variation to determine its clinical significance and whether ICU outcomes are also different at these times. It will also be essential to optimize our ability to monitor patients at all times of the day and keep lines of communication open despite varying hospital staffing.

## DISCLOSURE

The authors have no financial interest to declare in relation to the content of this article.
